# *In vitro* Activity of Antimicrobial Wound Dressings on *P. aeruginosa* Wound Biofilm

**DOI:** 10.3389/fmicb.2021.664030

**Published:** 2021-05-14

**Authors:** Ewa Klara Stuermer, Isabell Plattfaut, Michael Dietrich, Florian Brill, Andreas Kampe, Vanessa Wiencke, Anna Ulatowski, Maria Geffken, Julian-Dario Rembe, Ella Alexandrovna Naumova, Sebastian Eike Debus, Ralf Smeets

**Affiliations:** ^1^Department of Vascular Medicine, University Heart Center, University Medical Center Hamburg-Eppendorf (UKE), Hamburg, Germany; ^2^Institute of Virology and Microbiology, Faculty of Health, Centre for Biomedical Education and Research (ZBAF), Witten/Herdecke University, Witten, Germany; ^3^Dr. Brill + Partner GmbH, Institute for Hygiene and Microbiology, Hamburg, Germany; ^4^Institute for Transfusion Medicine, University Medical Center Hamburg-Eppendorf, Hamburg, Germany; ^5^Department of Vascular and Endovascular Surgery, Heinrich-Heine University Düsseldorf, Düsseldorf, Germany; ^6^Department of Biological and Material Sciences in Dentistry, Faculty of Health, School of Dentistry, Witten/Herdecke University, Witten, Germany; ^7^Department of Oral and Maxillofacial Surgery, University Medical Center Hamburg-Eppendorf, Hamburg, Germany; ^8^Department of Oral and Maxillofacial Surgery, Division of Regenerative Orofacial Medicine, University Medical Center Hamburg Eppendorf, Hamburg, Germany

**Keywords:** wound biofilm, wound dressing, antimicrobials, wound infection, PHMB, silver, octenidine dihydrochloride, iodine

## Abstract

The treatment of acute and chronic infected wounds with residing biofilm still poses a major challenge in medical care. Interactions of antimicrobial dressings with bacterial load, biofilm matrix and the overall protein-rich wound microenvironment remain insufficiently studied. This analysis aimed to extend the investigation on the efficacy of a variety of antimicrobial dressings using an *in vitro* biofilm model (lhBIOM) mimicking the specific biofilm-environment in human wounds. Four wound dressings containing polyhexanide (PHMB), octendine di-hydrochloride (OCT), cadexomer-iodine (C-IOD) or ionic silver (AG) were compared regarding their antimicrobial efficacy. Quantitative analysis was performed using a quantitative suspension method, separately assessing remaining microbial counts within the solid biofilm as well as the dressing eluate (representing the absorbed wound exudate). Dressing performance was tested against *P. aeruginosa* biofilms over the course of 6 days. Scanning electron microscopy (SEM) was used to obtain qualitative visualization on changes in biofilm structure. C-IOD demonstrated superior bacterial reduction. In comparison it was the only dressing achieving a significant reduction of more than 7 log_10_ steps within 3 days. Neither the OCT- nor the AG-containing dressing exerted a distinct and sustained antimicrobial effect. PHMB achieved a non-significant microbicidal effect (1.71 ± 0.31 log_10_ steps) at day 1. Over the remaining course (6 days) it demonstrated a significant microbistatic effect compared to OCT, AG and the control. Quantitative results in the dressing eluate correlate with those of the solid biofilm model. Overall, AG- and OCT-containing dressings did not achieve the expected anti-biofilm efficacy, while C-IOD performed best. Chemical interaction with the biofilms extrapolymeric substance (EPS), visualized in the SEM, and dressing configuration (agent concentration and release pattern) are suspected to be responsible. The unexpected low and diverse results of the tested antimicrobial dressings indicate a necessity to rethink non-debridement anti-biofilm therapy. Focussing on the combination of biofilm-disruptive (for EPS structure) and antimicrobial (for residing microorganisms) features, as with C-IOD, using dehydration and iodine, appears reasonably complementary and an optimal solution, as suggested by the here presented *in vitro* data.

## Introduction

Infected wounds, especially chronic wounds populated with biofilm, are one of the greatest challenges in modern wound care (Bowler et al., 2001; James et al., 2008; Demidova-Rice et al., 2012; Magill et al., 2014). Antimicrobial agents often fail in effective biofilm eradication (Percival et al., 2019b; Besser et al., 2020). This results from the symbiotic, multi-species society formed in biofilms by microorganisms, encasing themselves in a protective extrapolymeric substance (EPS), which acts as a shield against biochemical penetration by antimicrobial agents. Lateral resistance gene transfer between species and sub-species, dorment persister cells in the depth of biofilm and tissue as well as an effective biochemical diffusion barrier for active agents are further aspects contributing to the high resilience of microbial biofilms (Percival et al., 2015; Kirketerp-Møller et al., 2020). Even if an antimicrobial substance is capable of effectively eradicating a bacterial strain in its planctonic state, a deep enough penetration to achieve the same reductive efficacy, cannot be ensured in a complex biofilm scenario (Rembe et al., 2020).

Dressings and solutions containing silver, PHMB, octenidine or iodine have demonstrated favorable antimicrobial activity in several *in vitro* and clinical studies (Koburger et al., 2010; Storm-Versloot et al., 2010; Sibbald et al., 2011; Daeschlein, 2013; To et al., 2016; Dissemond et al., 2017). Anti-biofilm effects are often postulated alongside or were investigated in simple *in vitro* models, but are lacking validation in more complex test settings or clinical studies (O’Meara et al., 2000, 2013). To reduce the individual patient’s burden caused by chronic and infected wounds, prevention and treatment of pathogenic biofilm formation in wounds is a serious and relevant challenge in everyday clinical practice (Guest et al., 2017; Guest et al., 2018). Modern antimicrobial wound dressings represent promising therapeutic options in aiding to master this challenge (Daeschlein, 2013).

Mostly, *in vitro* evaluation of anti-biofilm activity is performed in models and settings less suited to sufficiently mimic a wound biofilm and the interactions with the human wound microenvironment. Such models and settings include simple single-species-biofilms, lower protein challenge than in clinical wounds, non-human matrix material (e.g., plastic or stainless steel surface) and one-dimensional biofilm structures (Brackman and Coenye, 2016; Shukla et al., 2020). In addition, most studies are not performed with dressings, but with antimicrobial solutions to prove the efficacy of a compound. However, solutions exhibit an entirely different biochemical and biophysical pattern of distribution, penetration and concentration in contact with a wounds micro-environment and biofilm residing in this environment, compared to wound dressings.

Thus, it is currently unclear whether the anti-biofilm activity of antimicrobial agents embedded in wound dressings is comparable to its antimicrobial liquid counterpart in the wound microenvironment. The presented work addresses this question *in vitro* using a modified human plasma-based biofilm model (lhBIOM) developed and described earlier by the authors (Besser et al., 2020; Rembe et al., 2020; Stuermer et al., 2021). The model mimics the condition of a wound biofilm by consisting of a bacteria-incorporating, matured, three-dimensional (3D) gel-like biofilm structure surrounded by bacteria-rich human plasma, which is similar to the composition of wound exudate in terms of protein content, carbohydrates, enzymes and bacterial breakdown products (Demidova-Rice et al., 2012; Stuermer et al., 2021).

## Materials and Methods

### Test Organism and Nutrient Solutions

*Pseudomonas aeruginosa* (DSM-939) was cultivated on casein/soy peptone agar plates (CSA) following standard procedures (DIN EN 13727). The first subculture was used for testing. The bacterial suspension was adjusted to a 0.5 McFarland standard (approx. 1.5 × 10^8^ cfu/mL) using a densitometer (Grant Bio^TM^ DEN-1B, Grant Instruments Ltd.; Cambs SG8 6GB, England). Bacterial counts were determined by spreading untreated controls of each experiment onto agar plates allowing exact calculations of surviving microorganisms as well as reduction rates.

### Test Wound Dressings and Preparation of Specimen

Five commercial dressings were investigated, four antimicrobial dressings containing either octenidine di-hydochloride (OCT), polyhexanide (PHMB), cadexomer-iodine (C-IOD) or ionic silver (AG) and an agent-free control dressing. Detailed data on dressing composition, contained active agent and concentration are displayed in Table 1. For all dressings, round pieces with a diameter of 2.2 cm (A = 3.8 cm^2^) were prepared in an aseptic manner fitting press-fit in one well of a standard 12-well-plate (Sarstedt, Nuembrecht, Germany). For analyses, pieces of each dressing were placed in 12-well plates containing the lhBIOM and surrounding bacteria-rich serum, to create a wound-like scenario with direct contact to the biofilm.

**TABLE 1:** Investigated dressings.

	**Manufacturer**	**Material**	**Active agent (concentration)**
UrgoClean^®^	Urgo GmbH, Chenôve, France	Cohesive polyabsorbent polyacrylate fibers; TLC wound matrix^®^; non-adhesive	None
Sorelex^®^	Contipro C, Dolní Dobrouč, Czech Republic	Permeable, gel-forming bioactive gauze, releases octenidine hydrochloride, and sodium hyluronat; non-adhesive	Octenidine di-hydrochloride (not indicated)
Suprasorb^®^ P + PHMB	Lohmann&Rauscher GmbH, Rengsdorf, Germany; Vienna, Austria	Semi permeable top film on polyurethane foam layer impregnated with and releasing PHMB; non-adhesive	Polyhexanide (0.25–0.65 mg/cm^2^)
UrgoClean^®^ Ag	Urgo GmbH, Chenôve, France	Cohesive polyabsorbent polyacrylate fibers; TLC wound matrix^®^; silver ion coating; non-adhesive	Ionic silver (0.50 mg/cm^2^)
IODOSORB^TM^ Dressing	Smith & Nephew GmbH, Hull, England	Beats of cadexomer (dextrin and epichlorhydrin) on gauze backing release iodine; non-adhesive	Iodine (0.90% w/w)

*Data of manufacturer, material, mechanism of action and contained substance concentration. Information has been obtained from the manufacturer.*

### Preparation of Leucocyte Rich Human Plasma Biofilm Model (lhBIOM)

The preparation of the lhBIOM was performed as described previously (Besser et al., 2020; Stuermer et al., 2021). In brief, fresh frozen plasma (FFP) of blood type AB (citrate buffered) and one LRS^®^ chamber of leukocyte apheresis (Trima Accel^®^; Terumo BCT Inc., Lakewood, United States) were obtained from the Institute for Transfusion Medicine (University Medical Center Hamburg-Eppendorf, Germany). Preparation of the lhBIOM was initiated on the same day of blood product donation. In brief, FFP was thawed, adjusted to 250 mL and the “immunocompetence” in form of leukocytes from the LRS^®^ chamber was added. The immune cells were obtained by using a special automated blood collection system for apheresis (Terumo BCT design, Trima Accel^®^ LRS^®^ Platelet, Plasma Set, REF number 82300; Terumo BCT Inc., Lakewood, United States), which removes nearly all leukocytes of the donor from the platelet sample, so that its concentration is equivalent to about 40 × 10^3^ leukocytes/μL. The content of one LRS^®^ chamber was placed in a tube, washed out with 3 mL of the FFP to remove any residual leukocytes, the wash-out added to the tube and centrifuged at 1,610 g. The layer of erythrocytes was gently removed and the remaining plasma-leukocyte mix added to the remaining FFP at room temperature. After gentle mixing, the bacterial suspension was added resulting in a final concentration of 1.5 × 10^6^ cfu/model. Next 18.26 μL CaCl_2_ (500 mM) per mL plasma was added to the bacteria-plasma-mix to induce coagulation. The resulting biofilm mixture was immediately transferred into 12-well plates (1.5 mL per model/well). Well plates were placed on a rotation shaker and incubated for 12 h at 60 rpm and 37.0°C to polymerize and form an extracellular matrix. After this period, stable biofilm discs with integrated test organisms resulted. Procedures of blood product collection are in accordance with “good clinical practice” standards and all donors gave their informed and written consent for the use of their blood products.

### Dressing Performance on *P. aeruginosa* Biofilm and Quantification of Bacterial Load

**FIGURE 1:**
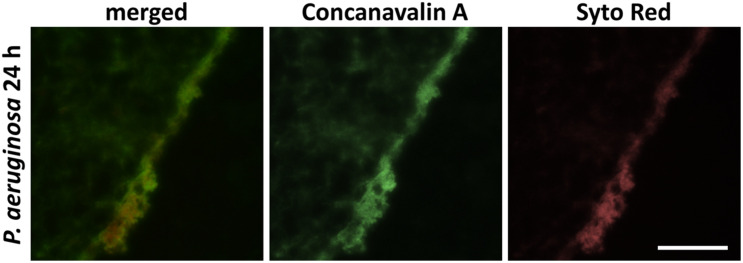
Immunohistochemical carbohydrates-staining of the glycocalyx of 24 h maturated biofilm produced by *P. aeruginosa*. Carbohydrates were detected with FITC-conjugated Con A, cellular and bacterial nucleic acids with SYTO Red staining (scale bar: 100 μm).

After 12 h of biofilm maturation in the lhBIOM (Figure 1), the test dressings were placed on the models as described above under “specimen preparation.” Treatment with dressings was performed for 1, 3, or 6 days without dressing change. After the specified treatment periods, wound dressings were carefully removed so that neither residues of the dressing remained on the model, nor biofilm substance was lost. Plastic beakers (50 mL) were filled with glass beads (D = 3–4 mm), so that the bottom was slightly covered. 10 mL neutralizer solution TLSNt-SDS (6% polysorbate 80, 6% saponin, 0.8% lecithin, 2% sodium dodecyl sulfat, 0.6% sodium thiosulfat in Aqua dest.) was added. The wound dressings were placed in the neutralizer solution. After shaking for 10 min at 200 rpm, extracts were plated out in 10-fold dilutions on CSA and incubated at 37°C under aerobic conditions for 48 h before quantification of colony forming units using a digital colony counter (NSCA 436000, VWR International GmbH; Germany).

Antimicrobial activity in the wells was neutralized by adding 300 μL of the neutralizing solution TLSNt-SDS to each well. Plates were subsequently placed on a rotation shaker for 5 min at room temperature for incubation of neutralizing agent. Subsequently, biofilm models were dissolved using bromelain (Bromelain from pineapple, Serva Electrophoresis GmbH; Heidelberg, Germany). Bromelain solution was prepared using 2.1 g powder, dissolved in 100 mL phosphate buffered saline (PBS) and 1.5 mL was added to each well containing a biofilm model. The biofilms were punctured with sterile pipette tips several times to ensure and accelerate complete dissolution of the model. After 3 h incubation (37°C; aerobic conditions) the biofilm models were completely dissolved with the exception of the iodine wound dressing models. These left insoluble residues. The microbial counts in the dissolved models were quantified in the same manner as the dressing eluates.

### Qualitative Evaluation by Scanning Electron Microscopy (SEM)

To visualize the antimicrobial effects of the wound dressings on biofilm morphology and structure, scanning electron microscopy (SEM) was performed. All dressings were carefully removed prior to further preparation of the models and antimicrobial activity were neutralized using the TLSNt-SDS neutralizing solution. Biofilm models were fixed with a glutaraldehyde/PVP-solution containing 2.5% glutaraldehyde, 2% polyvinylpyrrolidone (PVP) and 0.5% NaNO_2_ in 0.1 M cacodylate buffer for 1 h at 4°C. After washing three times (0.1M cacodylate buffer) they were prepared with liquid nitrogen to get freeze fracture fragments and stored in 0.1M cacodylate buffer. In order to stain the glycoxalyx, the samples were incubated for 18 h at RT in an arginine-HCl solution (solution with 2% arginine-HCL, glycine, sucrose and sodium glutamate). Next samples were rinsed again three times for 5 min with aqua dest. and subsequently stored for 5.5 h at RT in a mixture of 2% tannic acid and guanidine-HCL. After storage, samples were again rinsed once with aqua dest (5 min incubation) and three times with 0.1M cacodylate buffer (5 min incubation) and incubated overnight at 4°C. After overnight incubation, samples were placed in a 1% OsO_4_ solution for 30 min at RT followed by three rinsing steps with 0.1 M cacodylate buffer (10 min incubation) and stored again over night at 4°C. Last, samples were dehydrated in an isopropanol series (50, 70, 90, and 100%) for 15 min each followed by an acetone series (50, 75, and 100%) also each for 15 min. The drying step was completed by a final treatment in the critical point dryer (BAL-TEC AG, Balzers, Liechtenstein). With a sputter coater (BAL-TEC AG, Balzers, Liechtenstein), samples were sputtered with gold palladium and afterward analyzed by Zeiss Sigma VP SEM (Zeiss, Oberkochen, Germany) at 2 kV acceleration voltages using the in-lens and SE detectors.

### Statistical Analysis

Data is expressed as means ± standard error of the mean (SEM) based on triplicates derived from six different anonymous blood donors (FFP and leukocytes) at different time-points. Microbial reduction rates (in Δlog_10_ cfu/mL) were calculated and analyzed using the statistics program GraphPad PRISM (Version 9.0; GraphPad Software Inc., La Jolla, United States). For analysis of statistical significance a two-way repeated-measures ANOVA, followed by Tukey *post hoc* test for evaluation of multiple comparisons was applied. A *p*-value of *p* ≤ 0.05 was considered statistically significant.

## Results

### Quantitative Microbial Load in Wound Dressing Eluate

**FIGURE 2:**
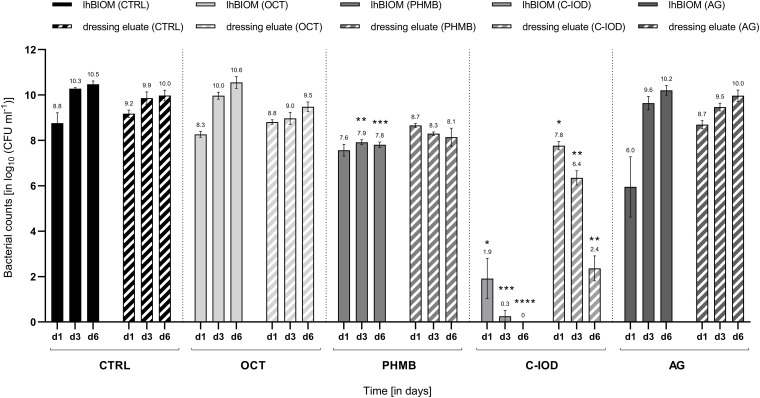
Comparison of the antimicrobial efficacy of wound dressings containing antimicrobial agents in the *P. aerugionosa* biofilm model lhBIOM. Reduction rates of bacteria (in log_10_ cfu/mL) are outlined after 1, 3, and 6 days of treatment with agent-free wound dressings (CTRL) and dressings containing octenidine (OCT), polyhexanide (PHMB), cadexomer-iodine (C-IOD), or ionic silver (AG). Bacterial content of the biofilm (solid bars) and in the dressing eluate (striped bars) are shown (values expressed as mean ± SEM. *p ≤ 0.05 vs. CTRL; **p ≤ 0.01 vs. CTRL, ***p ≤ 0.001 vs. CTRL, and ****p ≤ 0.0001 vs. CTRL).

Over the examined treatment course of a total of 6 days, the microbial counts in the wound dressing eluate followed a directly proportional pattern to the microbial load in the solid biofilm itself. Under treatment with octenidine di-hydrochloride (OCT) and ionic silver (AG), a gradual increase of microbial counts on days 1, 3 and 6 were observed (Figure 2). Such increasing microbial counts also occurred in the control dressing (CTRL) with no antimicrobial agent, consequently showing no anti-biofilm activity of OCT and AG on the wound dressing eluate. PHMB demonstrated a statistically non-significant decrease in microbial counts of the eluate on day 1 (0.62 ± 0.16 log_10_ steps) compared to initial counts. Subsequently, the PHMB dressing maintained the level of microbial counts over the remaining course of 6 days compared to the increasing counts in the control dressing (Figure 2), demonstrating a bacteriostatic effect.

On day 1, treatment with the C-IOD dressing resulted in a statistically non-significant reduction of 1.51 ± 0.41 log_10_ steps compared to initial bacterial counts in the dressing eluate. After 3 and 6 days of treatment, C-IOD achieved a significant reduction of microbial counts in the eluate compared to initial counts of 2.93 ± 0.29 log_10_ steps (*p* = 0.0207) and 6.92 ± 0.58 log_10_ steps (*p* = 0.0154), respectively. Compared to the control dressing this accounts for a significantly reduced bacterial burden of 1.41 ± 0.24 log_10_ steps on day 1 (*p* = 0.0254), 3.51 ± 0.42 log_10_ steps on day 3 (*p* = 0.0069) and 7.62 ± 0.60 log_10_ steps on day 6 (*p* = 0.0077; Figure 2).

### Anti-biofilm Activity of Antimicrobial Wound Dressings on the lhBIOM

Regarding the antimicrobial effect on bacteria within the biofilm model, the results demonstrated an overall similar trend for wound dressings and dressing eluate (Figure 2). Treated with an OCT-containing dressing over a 6-day-period, microbial counts within the lhBIOM showed a continued increase with no observable antimicrobial effect. The AG-containing dressing demonstrated similar results, with the exception of an initial significant decrease in microbial counts on day 1 of 3.33 ± 1.50 log_10_ steps (*p* = 0.013) compared to initial counts. On day 3 however the initial reductive effect had worn off and microbial re-growth above initial counts had occurred.

After an initial non-significant reduction on day 1, the PHMB-impregnated dressing maintained a prolonged bacteriostatic effect. This was observed for the biofilm itself as well as in the dressing eluate. PHMB reduced the initial bacterial counts by 1.71 ± 0.31 log_10_ steps, subsequently keeping it at the reduced level over the course of 6 days without occurring re-growth (Figure 2). Compared to the control dressing, PHMB obtained a significant reduction in bioburden of 2.36 ± 0.12 log_10_ steps on day 3 (*p* = 0.0028) and 2.66 ± 0.19 log_10_ steps on day 6 (*p* = 0.0010) in the lhBIOM. The reduction on day 1 compared to the control dressing, however, was not statistically significant.

The C-IOD containing dressing showed the highest anti-biofilm activity. On day 1, bacterial counts in the biofilm were significantly reduced by 7.37 ± 0.99 log_10_ steps (*p* = 0.0387) compared to initial counts. On day 3 the initial bacterial load was reduced by 9.03 ± 0.45 log_10_ steps (*p* = 0.0054) and on day 6 no quantifiable microrganisms were retrieved, representing a complete reduction of initial microbial counts (9.28 ± 0.24 log_10_ steps; *p* = 0.0015; Figure 2).

### Visual Analysis of Dressing Effects on the lhBIOM Using Scanning Electron Microscopy (SEM)

**FIGURE 3:**
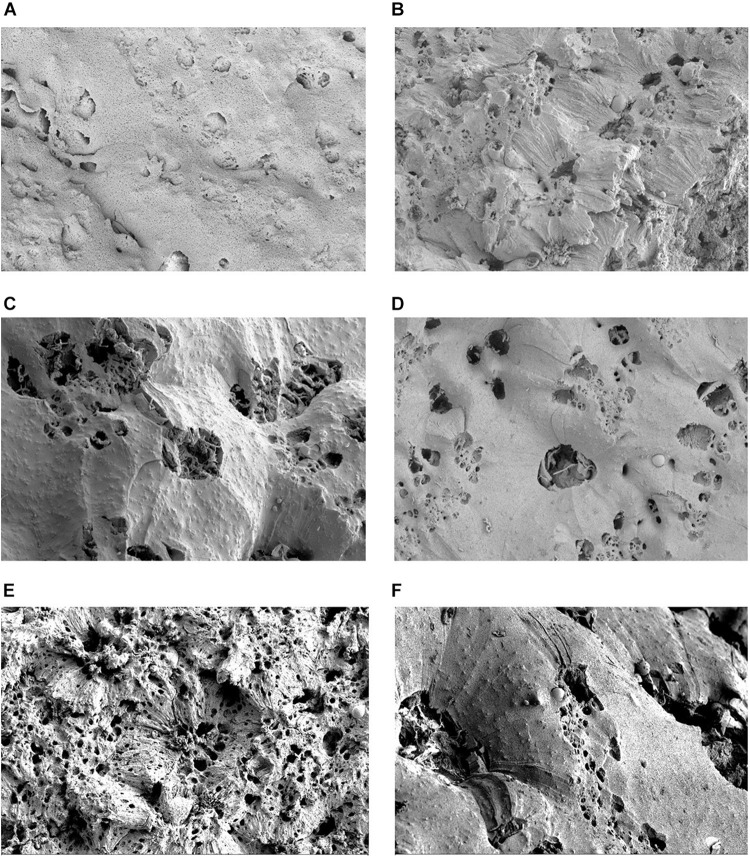
Scanning electron microscopy (SEM) visualization of *P. aerugionosa* biofilm surface (lhBIOM) after 3 days under control conditions **(A)** and after treatment with commercial wound dressings: **(B)** polyurethane dressing with no active agent; **(C)** with octenidin (OCT); **(D)** with polyhexanide (PHMB); **(E)** with cadexomer-iodine (C-IOD) and **(F)** ionic silver. The untreated biofilm model **(A)** shows a densely connected surface structure. After treatment with cadexomer-iodine **(E)**, surface structure appears rugged and “broken-open” with several holes as potential new entry points for iodine. After treatment with OCT **(C)** and PHMB **(D)** biofilm surface remains more dense though several holes are visible. Silver **(F)** induced the least changes.

SEM analysis of *P. aeruginosa* biofilm treated with wound dressings for 3 days (Figure 3) showed a distinct modification of the biofilm surface for all models compared to the untreated biofilm (Figure 3A). Whereas with drug-free wound dressings only an undulating fissuring with an almost intact surface was visible (Figure 3B), those containing antimicrobials induced an increased porosity. This was most prominent in the cadexomer-iodine dressing (Figure 3E). The former induced a shotgun-like change in the biofilm surface reflecting the penetration of iodine from the cadexomer beads into deeper layers. The OCT- (Figure 3C) and PHMB-containing wound dressings (Figure 3D) showed a smooth surface with multiple isolated but rather big holes and their structural appearances were quite similar. The AG-containing dressing seemed to induce a more pronounced roughening of the biofilm surface with, however, fewer entry holes and an overall lower porosity (Figure 3F).

## Discussion

In guidelines, local antimicrobials such as different silver formulations, polyhexanide (PHMB), octenidine, dihydrochloride or iodine are rated as equally efficient (AWMF, 2012). Consensus about advantages and disadvantages in direct comparison, recommendations and independent analyses of efficacies in complex test scenarios or clinical conditions are, however, rare (Kramer et al., 2018). Even though several studies addressed this issue, most concluded that further analyses are mandatory to gain evidence-based recommendations for the daily use of antimicrobial dressings (Storm-Versloot et al., 2010; Daeschlein, 2013; Forster and Pagnamenta, 2015; Wu et al., 2015; Norman et al., 2016). Using a complex 3D model designed to mimic the micro-environment of a human wound biofilm (lhBIOM), composed of human material (plasma, platelets and leukocytes), this study aimed to extend the knowledge-base on efficacy profiles of antimicrobial wound dressings in a more transferable, “closer-to-reality” test scenario (Besser et al., 2020; Rembe et al., 2020; Stuermer et al., 2021). In this model, the incorporated bacteria encounter not only a milieu similar to the wound exudate (Loeffler et al., 2013), but also a certain immunological competence represented by leukocytes, so a biofilm matrix has to be produced under the challenge of a donor’s immunocompetence (Figure 1).

The obtained results of the investigated wound dressings regarding antimicrobial efficacy partly contrast what is currently considered best-practice knowledge and has been previously reported. In case of the iodine-containing product (C-IOD), a distinct and continuous antimicrobial effect was observed in this study with a successive microbial reduction within 3 days (Figure 2), confirming previous *in vitro* and clinical studies (Phillips et al., 2015; Fitzgerald et al., 2017; Roche et al., 2019). It should be emphasized, however, that under treatment with the cadexomer-iodine dressing, the biofilm model was not completely dissolved in bromelain. This most likely arises from the proposed dehydrative effect caused by the cadoxmer agent (Akiyama et al., 2004; Fitzgerald et al., 2017). Judging by the reductive pattern in the solid biofilm model (lhBIOM), it seems, that the cadexomer-proportion of the dressing degrades and increases the porosity of the biofilm structure. Combined with iodine it acts partly lethal on exposed bacteria, while partly binding microorganisms within the dressing. This offers an explanation for the higher remaining microbial counts within the eluate on day 1 (Figure 2): Released microorganisms from the degraded biofilm structure are initially bound and subsequently reduced by the continued release of iodine. The simultaneous continued, slow release of iodine molecules into the degraded biofilm also reaches deeper structures and works against dorment bacteria. This approach is also supported by the SEM images, showing a failure of the closed protective EPS shield due to the impact of cadexomer-iodine.

Evaluations regarding the anti-biofilm activity of polyhexanide-containing wound dressings (PHMB) are rarely found in the literature (Huebner and Kramer, 2010; Huebner et al., 2010; Lenselink and Andriessen, 2011; Davis et al., 2017; Kramer et al., 2018); for the octenidine-containing wound dressing (OCT) no data at all could be found. Most efficacy assumptions and statements are transferred from analyses performed on microorganisms in a planktonic state or from evaluations of the antiseptic solution counterparts. Regarding the PHMB dressing, a reductive efficacy (~2 log_10_ steps) with a subsequent bacteriostatic effect could be demonstrated in this work, presumably due to the comparably high concentration of PHMB (0.65 mg/cm^2^) embedded in and released by the wound dressing (Figure 2 and Table 1). These results are in accordance with the results of earlier analyses of our working group, demonstrating a good efficacy for PHMB-containing antiseptics and antimicrobial irrigation solutions (Brackman and Coenye, 2016; Rembe et al., 2020). However, this positive quantitative effect is not reflected in the SEM images, since only slightly changes to the surface of the biofilm can be observed.

For the here tested silver-containing wound dressing (AG), the expected high antimicrobial efficacy could not be verified. While several *in vitro* evaluations in planktonic or simpler biofilm models demonstrated a good antimicrobial and anti-biofilm efficacy (O’Meara et al., 2000; Desroche et al., 2016; Percival et al., 2019b), these results could not be reproduced in a more complex *in vitro* biofilm scenario used here (Figure 2). Similar discrepancies and debates arise from various previous clinical studies of critically colonized or infected wounds, resulting in the necessity for further investigations (Lo et al., 2009; O’Meara et al., 2013; Dissemond et al., 2017). Even though a initial bacterial reduction was observed after 1 day of treatment, re-growth occurred over the following course of 6 days treatment, ultimately displaying similar microbial counts as the control dressing. Analyzing the eluate of the silver dressing, as a surrogate for the absorbed microorganism-containing wound exudate, there was also no reduction of the microbial load. These findings are contrary to previous descriptions by Desroche et al. (2016), stating that it “reduces the bacterial population and the biofilm of *P. aeruginosa* and MRSA up to 4 log steps within 24 h for 7 days” (collagen I-coated surface with no human components). The AG-impregnated wound dressing contains ionic silver coated to the specific surface matrix in a comparably low concentration (0.50 mg/cm^2^; Table 1). Besides the low concentration of the active agent, the composition of the milieu seemed to impede silver to exert its full antimicrobial effect. The relevance of its chemical structure has already been proven in previous studies. The loss of efficacy was pointed out for nanocrystalline silver (Gnanadhas et al., 2013; Rembe et al., 2018) more than for ionic silver, however, interactions with the wound microenvironment such as pH value or protein content have been described for both several times (Hirsch et al., 2011; Kapalschinski et al., 2013; Wiegand et al., 2015; Rembe et al., 2018). This emphasizes the need for human-adapted analyses *in vitro*, as the here tested dressing containing ionic silver demonstrates an overall loss of antimicrobial efficacy in a complex, protein-rich, human-adapted microenvironment.

Surprisingly, the octenidine-impregnated dressing showed no antimicrobial or anti-biofilm efficacy in the performed experiments, while the antiseptic solution tested in a similar complex model (hpBIOM) in previous studies, demonstrated the highest efficacy compared to other antimicrobial solutions (Besser et al., 2020; Rembe et al., 2020). In many previous studies, both against planktonic bacteria as well as biofilm, the active agent of the disinfectant Octenisept^®^ has repeatedly proven to be highly effective (Brackman and Coenye, 2016; Rembe et al., 2020), even though the onset of its full efficacy was shown to be delayed in complex biofilm scenarios (up to 48 h). The missing antimicrobial activity of the OCT dressing might therefore be attributed to the dressing configuration with a retained release of the active agent into the wound (here biofilm model) or an insufficient amount of active agent embedded and released to achieve an impact. Unfortunately, the exact amount of octenidine di-hydrochloride in the dressing is not provided in the literature or by the manufacturer. However, the question of substance concentration has shown differences in antimicrobial dressing performance in previous studies, with lower concentrations yielding lower reduction rates (Rembe et al., 2018). Another restrictive factor of the dressing configuration might be the combination of octenidine, dihydrochloride and hyaluronic acid in the wound contact layer, which upon contact with wound exudate forms a gelling structure. This can lead to an “entrapment” of the active agent OCT in the gelling layer with the main antimicrobial activity exerted on microorganisms absorbed with the wound exudate into this layer and therefore low release of the agent into the wound and biofilm. Additionally, in the here tested dressing, only octenidine di-hydrochloride is embedded, in contrast to the highly effective liquid antiseptic Octenisept^®^, which contains a combination of octenidine di-hydrochloride and phenoxyethanol. The additive phenoxyethanol, however, exerts additional antimicrobial effects and further contained additives as well as alcohols have been specifically attributed with biofilm degrading properties (Percival et al., 2017a,b, 2019a), therefore offering another explanatory factor for the missing antimicrobial and anti-biofilm efficacy in the presented results. The question whether a daily or 2-day dressing change, would enhance the performance of the less effective test dressings (AG and OCT) cannot be conclusively answered herein. In relation, however, the more effective dressings (PHMB and C-IOD) would expectedly also profit from a more frequent change regimen, therefore not altering the comparative performance.

The aspects regarding active substance concentration and physicochemical release patterns as well as specific interactions due to dressing and microenvironment composition are transferable and applicable to all here tested wound dressings. Supposedly, the specific constellation of such factors for individual dressings dictate the overall antimicrobial and anti-biofilm efficacy observed in the presented results. Here, the polyhexanide-containing (PHMB) and especially the cadexomer-iodine containing dressing (C-IOD) proved to be most active against *P. aeruginosa* biofilm compared to other antimicrobial wound dressing formulations. However, only cadexomer-iodine achieved an actual relevant bactericidal effect. This is also reflected in the SEM showing an increased porosity and a distinct alteration of the biofilm surface pattern. Similar results were observed in earlier research approaches regarding its anti-biofilm activity (Phillips et al., 2015). As mentioned earlier, the combined cadexomer-iodine dressing exerts its effect by two main aspects: Cadexomer directly destroys biofilm structures by collapsing the bacterial glycocalyx (EPS) which is composed of 99% water (Lapping-Scott et al., 2014) by dehydration through water absorbtion, while iodine as an antimicrobial agent can subsequently eliminate bacteria (here *P. aeruginosa*) released and exposed from the damaged biofilm structure (Akiyama et al., 2004).

Naturally, the presented results are limited as to the fact that they are *in vitro* results, calling for a careful consideration in terms of translation to clinical situations. Due to the experimental setup using a complex, human material based approach, the evaluations have been moved one step closer to clinical reality, however, still need to be interpreted with caution and in relation. The here used model allows for far more precise interpretations of results in terms of transferability into reality than simpler models. Still, even more complex test settings (incorporating human cell lines and three-dimensional tissue structures) will be required in the future to even better interpret complex efficacy interactions of antimicrobial products. The same goes for well-designed, sufficiently powered randomized controlled clinical trial to back *in vitro* findings and establish long-sought evidence-based clinical guidelines. The present results, however, show that these are urgently needed to clarify indications and support correct choice of wound care products.

Further limitations include the intentional disregard of the negative aspect of cytotoxicity potentially caused to the regenerating wound by excessive release of antimicrobial substances from wound dressings, as well as the evaluation of only one dressing per agent group with only a single combination of substance concentration and dressing formation per group investigated. Cytotoxic aspects were intentionally not addressed in this work due to focus being placed on bactericidal effects against biofilms, which untreated would exert potentially more harm to the healing wound than a confined cytotoxic impact derived from an antimicrobial treatment.

Finally, questions regarding the relevance of physicochemical release patterns and the specific composition and placement of the active agent within the dressing would be better addressed comparing differently manufactured dressings. However, in most cases (OCT, C-IOD, and PHMB) only very limited or even no further dressings containing the specific antimicrobial agents are commercially available and therefore need to be specifically manufactured as prototypes. This represents a continuous future endeavor in the field of material science, to identify and reliably validate the best combination of wound dressing material and active antimicrobial agent.

## Conclusion

In this *in vitro* study the challenge a wound biofilm poses for antimicrobial agents becomes evident once again. The iodine- and polyhexanide-containing dressings perform as expected with a high bactericidal effect of C-IOD and a sustained bacteriostatic effect of PHMB over the course of 6 days even though not all surviving bacteria were counted due to incomplete lysis of the biofilm under C-IOD. The silver- and octenidine-containing wound dressings on the contrary did not show a bactericidal or bacteriostatic activity in the employed complex biofilm model (lhBIOM). In all wound dressings, the overall composition of the dressing, the concentration of the active substance and the form of interaction with the microenvironment are postulated to be crucial factors. For future anti-biofilm treatment strategies, especially dressings should be sought out, that exhibit biofilm/EPS degrading as well as antimicrobial properties, either in a single active substance or combinations. Therefore, biocompatible active agents or additives, that are readily and continuously released into the wound to interact with the biofilm need to be further investigated.

## Data Availability Statement

The raw data supporting the conclusions of this article will be made available by the authors, without undue reservation.

## Author Contributions

ES, FB, and RS designed the study. IP, VW, AU, MD, and MG carried out the experiments. ES, J-DR, AK, and RS analyzed the results and performed statistical analysis. IP, MD, and EN made the REM pictures and figures. ES, J-DR, SD, and RS drafted the manuscript. All authors contributed to the article and approved the submitted version.

## Conflict of Interest

The authors declare that the research was conducted in the absence of any commercial or financial relationships that could be construed as a potential conflict of interest.
